# A Predictor of Pathological Complete Response to Neoadjuvant Chemotherapy Stratifies Triple Negative Breast Cancer Patients with High Risk of Recurrence

**DOI:** 10.1038/s41598-019-51335-1

**Published:** 2019-10-16

**Authors:** Marcia V. Fournier, Edward C. Goodwin, Joan Chen, John C. Obenauer, Susan H. Tannenbaum, Adam M. Brufsky

**Affiliations:** 1 Bioarray Genetics Inc, 400 Farmington Ave, Farmington, CT 06032 USA; 2 Rancho Biosciences, 16955 Via Del Campo #220, San Diego, CA 92127 USA; 3 Division of Hematology/Oncology, UCONN Health Center, 263 Farmington Ave, Farmington, CT 06030 USA; 4 Division of Hematology/Oncology, University of Pittsburg School of Medicine, 300 Halket St # 4628, Pittsburgh, PA 15213 USA

**Keywords:** Predictive markers, Breast cancer, Cancer genomics

## Abstract

We developed a test to predict which patients will achieve pathological complete response (pCR) to neoadjuvant chemotherapy (NAC) and which will have residual disease (RD). Gene expression data from pretreatment biopsies of patients with all breast cancer subtypes were combined into a 519-patient cohort containing 177 TNBC patients. Two RNA classifiers of 16 genes each were sequentially applied to the total cohort, classifying patients into 3 distinct classes. The test performance was further validated in an independent 304-patient cohort. The test accurately identified 70.5% (79/112) of pCR and 83.5% (340/407) of RD patients in the total population, and 75.0% (45/60) of pCR and 75.2% (88/117) of RD patients in the TNBC subset. For the independent cohort, the test identified 91.5% RD patients in the total population and 86.2% RD patients in the TNBC subset. However, the identification of pCR in both total and TNBC population are as low as 21.1% and 30%, respectively. The TNBC RD patients were subdivided by our classifiers, with one class showing significantly higher levels of Ki67 expression and having significantly poorer survival rates than the other classes. This stratification of patients may allow predicted residual disease classes to be assigned an alternative therapy.

## Introduction

Triple-negative breast cancer (TNBC), characterized by lack of expression of the estrogen (ER), progesterone (PgR), and erb-b2 receptor tyrosine kinase 2 (HER2) receptors, is a particularly problematic form of breast cancer due to aggressive growth, high recurrence rates and poor long-term survival^1,2^. TNBC represents 15–20% of newly diagnosed breast cancers in the United States^3^. Achieving pathological complete response (pCR) to neoadjuvant chemotherapy (NAC) is a surrogate marker and predictor of long-term outcomes, especially for TNBC^4–11^. Thus, NAC can allow for an early evaluation of the effectiveness of systemic therapy. Since pCR is correlated with prediction of 5-year disease free survival, biomarker development in this setting can establish efficacy which can then be utilized in both the neoadjuvant and adjuvant settings. In addition, only about 20% of breast cancer patients achieve pCR^11^, causing unnecessary morbidity for the other 80% receiving high-toxicity treatment with limited benefit. Predicting which patients will have pCR or residual disease (RD) provides physicians with an opportunity to improve treatment planning with more aggressive or novel treatments, while preventing overtreatment in populations expected to achieve pCR with the standard of care.

pCR predictors have been proposed, but they either did not achieve the necessary levels of positive and negative predictive values for clinical utility, suffered from small sample sizes, lacked validation data, or were not applicable to TNBC^12–21^. While effective molecular tests exist to guide treatment for estrogen receptor (ER) or HER2+ tumors, there are no tests in clinical use to stratify TNBC. High levels of tumor infiltrating lymphocytes (TILs) have shown a correlation with increased pCR rates in TNBC, in that 31% of the low-TIL patients achieved pCR compared to 50% of the high-TIL patients^22^. But this correlation is not strong enough to use as a predictive model, because it would predict pCR for all high-TIL tumors and would be wrong for 50% of the patients. Patients with grade 3 tumors, containing cells morphologically different from healthy cells, were found to have higher pCR rates in platinum-based neoadjuvant therapy than patients with lower-grade tumors^23^. However, the reported odds ratio of this finding was 1.73, which converts to a probability of 63.4%, and this would also be its accuracy if used as a predictive test. Gene expression profiling has been used to define four TNBC subtypes with significant differences in their pCR rates^15^. But again, even the subtype most associated with pCR contained only 41% of the patients that achieved pCR, which is still not a clinically useful predictor. There is a strong unmet need for a predictor of response to neoadjuvant chemotherapy for TNBC patients that is sufficiently accurate to use in treatment decisions.

We developed a test called BA100 to predict which patients are likely to achieve pCR or RD to standard NAC using gene expression profiling of 325 novel biomarkers that are associated with non-malignant breast epithelial cell organization and that correlate with breast cancer clinical outcomes^24–26^, 23 TNBC-related genes^27^, and a unique and proprietary machine learning algorithm to select and rank informative genes. While the 325 genes are mainly involved in pathways such as proliferation, DNA repair, cell survival, metabolisms, cell migration and adhesion, two genes in the 23 TNB-related genes are likely involved in cell immunity. One gene is *HLA-DPA1*, which encodes a major histocompatibility complex, class II, DP alpha 1 protein. This protein is mainly expressed in antigen presenting cells (APC) including macrophages, dendritic cells, as well as B lymphocytes as hetero-dimers with HLA-DPB, and plays an essential role in human immune system by presenting the extracellular peptides to T cells^28^. The other gene *VTCN1*, encoding V-set domain containing T cell activation inhibitor 1, belongs to the B7 costimulatory protein family. Proteins in this family are also present mostly on APCs, and interact with ligands that bind to receptors on cytotoxic T cells. VTCN1 was found to negatively regulate cell immune response, and expression of VTCN1 negatively correlates with patient’s clinical outcome^29^. The results show that in addition to stratifying patients into pCR and RD classes, the BA100 test also identifies a third class of RD patients with worse survival than the others. We characterized these three classes of patients to see if some are associated with higher residual cancer burden (RCB), which is clinically similar to pCR/RD classification; whether they correspond to some of the breast cancer subtypes defined by the PAM50 molecular classifier; and whether they differ in Ki-67 or androgen receptor (AR) expression, which are known to impact the aggressiveness of breast cancers. We also compare our BA100 predictor to a previously published one called DLDA30^21,30^.

## Methods

### Patients and data sets

A patient data set of Affymetrix Human Genome U133A GeneChip® Array gene expression with associated outcomes and treatment data was constructed from three patient cohorts with stage II-III invasive breast cancer treated with standard NAC incorporating a taxane, an anthracycline, and cyclophosphamide (AC-T), or additionally 5-fluorouracil (T-FAC). The I-SPY1 (Investigation of Serial Studies to Predict Your Therapeutic Response with Imaging and Molecular Analysis) trial^5^ contained breast cancer patients of all subtypes who received AC-T NAC, with HER2+ patients also receiving trastuzumab, while two studies from NCBI’s Gene Expression Omnibus (GEO), GSE25055 and GSE25065^13^, contained patients receiving T-FAC. After duplicates and samples with missing data were removed, a combined cohort of 519 patients remained. The cohort included 56.1% of patients with ER+/HER2- and 34.1% with TNBC, based on the annotated hormone receptors and HER2 status provided in the studies used. Suppl. Table 1 shows TNBC patient demographics and histopathological information. In short, 85.3% of TNBC patients were grade 3, with 33.9% achieving pCR, and 66.1% had residual disease.

An independent external validation set of 304 patients was constructed from GEO studies GSE20194^31^, GSE20271^21^, and GSE32646^32^ by removing duplicate patient entries between each other and the 519-patient training and testing cohort. Patient demographics are shown in Supplemental Table 2. The pCR rate for the 304-patient external validation cohort was 18% for all patients and 24% in TNBC.

### Expression data preparation

Affymetrix’s CEL files for the 519-patient dataset were downloaded from GEO, imported into R using the BioConductor Affymetrix package, processed using the BioConductor mas5 package, subjected to batch correction using ComBat from the sva R package, and quantile normalized. CEL files for the 304-member data set were treated identically with the exception that they were normalized using quantiles obtained from 519-patient training and test set, and no batch correction was performed, in order to simulate real-world single-sample testing.

### Development of RNA-biomarker classifiers

The original patient cohort was divided into a training set of 80% patients and a testing set of 20% patients. The training and testing sets had similar fractions of pCR and RD patients, and similar representations of ER, Her2, and PGR status. The training cohort was further subdivided into temporary 80%/20% subsets as part of thousands of rounds of model development, with each round having different samples selected from the pCR and RD groups. Starting with the 325 genes described above^24–26^, a proprietary algorithm based on backward regression general linear modeling (BRGLM) was used to select a smaller set of genes with the greatest predictive power. This resulted in 16 genes that were used to fit a linear regression model. To reduce the numbers of false positives detected among TNBC patients, we added genes from a TNBC signature^27^, repeated the modeling process using only patients that were predicted to achieve pCR by the first model, and developed a second 16-gene classifier. The use of two sequential classifiers containing different genes allowed us to combine a general predictor for all disease subtypes with a second predictor that improves the classification of TNB patients. It also made possible the identification of the third class of RD patients with worse prognosis. This modeling method has also been described in two conference proceedings. The sequential 16-gene/16-gene classifiers were applied to the 519-patient cohort and the 304-patient external validation cohort.

**Figure 1:**
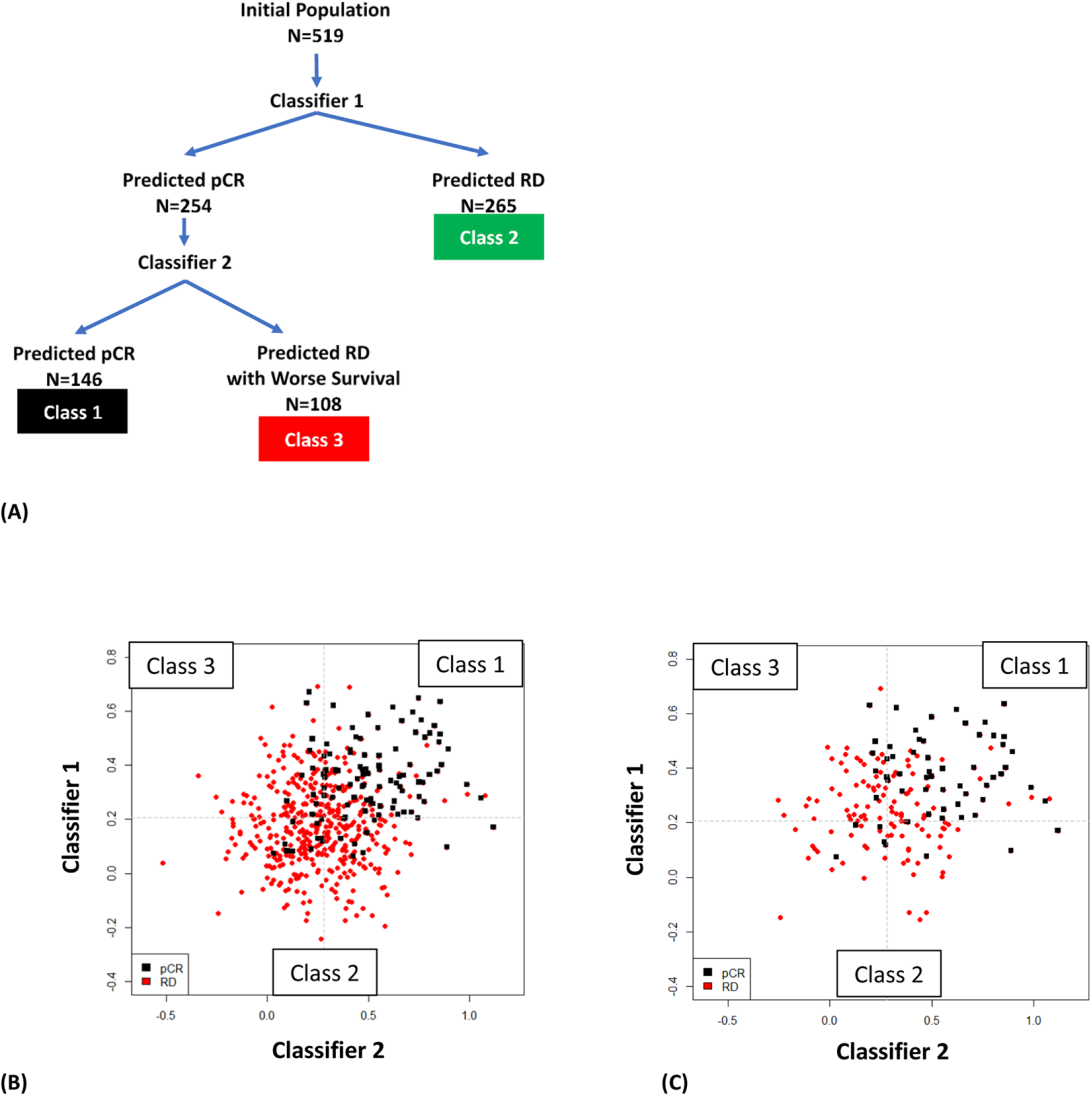
Description of BA100 test. (**A)** A diagram showing the flow of patients from the 519-member data set through the two classifiers of BA100 and resolution into 3 classes based on unique gene profiles. “N” represents the number of patients stratified at each step. (**B)** Output of the BA100 scores for the total population of 519 patients. The black squares represent patients achieving pCR, the red squares those with RD and the dashed lines the cutoff values above which pCR is predicted. Scores from Classifier 1 are on Y-axis and Classifier 2 on the X-axis. The Class 1 patients are those that are predicted pCR by both classifiers in the upper right quadrant, Class 2 those predicted RD by Classifier I (bottom half) and Class 3 those that Classifier 1 predicted pCR while Classifier 2 predicted RD (upper left quadrant). (**C)** Output of the BA100 scores for the TNBC population of 177 patients. Description as in (**B)**.

Classifier 1 stratified patients into the first predicted RD group (Class 2). Classifier 2 was then applied to stratify the remaining population into a predicted pCR (Class 1) and a second group of predicted RD (Class 3) (Fig. 1A). The genes comprising the classifiers are shown in Supplemental Table 3 along with their coefficients, intercepts, and threshold values above which pCR is predicted.

### Kaplan-Meier plots

Distant recurrence-free survival (DRFS) data was available for a maximum of 10 years in the 518-patient cohort (1 TNBC patient from the 519-member data set lacked unambiguous survival data and was excluded). Censoring was performed as indicated within the public data files. Standard Kaplan-Meier curves were generated using R packages and the statistical significance was determined by the Cox proportional hazard model with Wald and logrank test.

### Comparisons of tumor characteristics

Classifications by PAM50, residual cancer burden (RCB), and a pre-defined 30-gene predictor (DLDA30)^21,30^ were annotated for most of the 519-patient data set and were tallied and presented as column plots. The values for androgen receptor (AR) and Ki-67 gene expression were extracted from the expression data, segregated by BA100 class and presented as box plots. The Pearson chi-squared test of independence and t-tests were used to calculate associations of RCB, PAM50 subtype, AR expression, and Ki-67 expression with BA100 classes.

### Ethics approval and consent to participate

The study does not involve human subjects.

## Results

### Stratification of patients

Scores from the sequential application of our two classifiers (Fig. 1A) are shown as 2-dimensional scatter plots for the total population and 177 TNBC patients, respectively in Fig. 1B,C, with pCR concentrated in the upper right quadrant corresponding to the predicted positive (class 1). Conversely, the scores for RD patients are predominately scattered over the other 3 quadrants comprising classes 2 and 3.

The results of applying BA100 to stratify patients into pCR (Class 1) and RD (Classes 2 and 3 combined) in the 519-patient data set are shown in Table 1. The pCR rates of each population are shown to range from 10.3% for ER+/HER2- to 48.5% in HER2+ patients. The test correctly stratified 70.5% (79/112) of pCR and 83.5% (340/407) of RD patients in the total population, and 60.8% (45/74) of pCR and 85.4% (88/103) of RD patients in the TNBC subset. The overall accuracy was 80.7% in the total cohort and 75.1% in the TNBC subset.

**Table 1:** The top section shows results of BA100 stratification on the 519-patient data set for four subtypes and total population.

	519 Member Discovery and Testing Data Set (16/16 model)									
	pCR	BA100 Performance Metrics								
		PPV	NPV	Sensitivity	Specificity	TP	FP	TN	FN	Total
ER+HER2-	10.30%	39.20%	95.80%	66.70%	88.10%	20	31	230	10	291
TNBC	33.90%	60.80%	85.40%	75.00%	75.20%	45	29	88	15	177
HER2+	48.50%	69.20%	65.00%	56.30%	76.50%	9	4	13	7	33
ER-HER2-PR+	33.30%	62.50%	90.00%	83.30%	75.00%	5	3	9	1	18
Total Population	21.60%	54.10%	91.20%	70.50%	83.50%	79	67	340	33	519
	**pCR**	**DLDA30 Performance Metrics**								
		**PPV**	**NPV**	**Sensitivity**	**Specificity**	**TP**	**FP**	**TN**	**FN**	**Total**
TNBC	35.20%	37.50%	76.00%	88.20%	20.20%	45	75	19	6	145
	**304 Member External Validation Data Set (16/16 model)**									
	**pCR**	**BA100 Performance Metrics**								
		**PPV**	**NPV**	**Sensitivity**	**Specificity**	**TP**	**FP**	**TN**	**FN**	**Total**
ER+HER2-	6.80%	20.00%	93.80%	11.10%	96.80%	1	4	120	8	133
TNBC	23.50%	40.00%	80.00%	30.00%	86.20%	6	9	56	14	85
HER2+	34.10%	41.70%	67.10%	17.90%	87.00%	5	7	47	23	82
ER-HER2-PR+	0.00%	0.00%	100.00%	NA	75.00%	0	1	3	0	4
Total Population	18.80%	36.40%	83.40%	21.10%	91.50%	12	21	226	45	304

The second through fifth columns shows the rate of actual pCR in the indicated population prior to testing, and the positive predictive value (PPV), negative predictive value (NPV), sensitivity, and specificity of the BA100 model’s predictions. The actual numbers of patients in each predicted population are shown as true positive (TP), false positive (FP), true negative (TN) and false negative (FN), where a positive prediction means pCR, and a negative prediction means RD. Immediately below the BA100 data we show the results of the DLDA30 predictor for the 145 TNBC patients where such data was available. The bottom panel shows the starting populations and results of BA100 application to the 304-patient external validation data set.

The data set included values for the DLDA30 predictor for 145 of the 177 TNBC patients and the resulting test predictions are shown in Table 1 for comparison. DLDA30 correctly stratified 37.5% (45/120) of the pCR and 76.0% (19/25) of the RD patients, with an overall accuracy of 44.1%. BA100 outperforms DLDA30 on each of these measures.

### BA100 stratifies TNBC patients into distinct classes of patients

Given that unique gene expression profiles from 2 independent classifiers defined biologically distinct classes of patients (BA100 Class 1–3), we examined if the classes correlated with clinical outcomes and other biomarkers.

**Figure 2:**
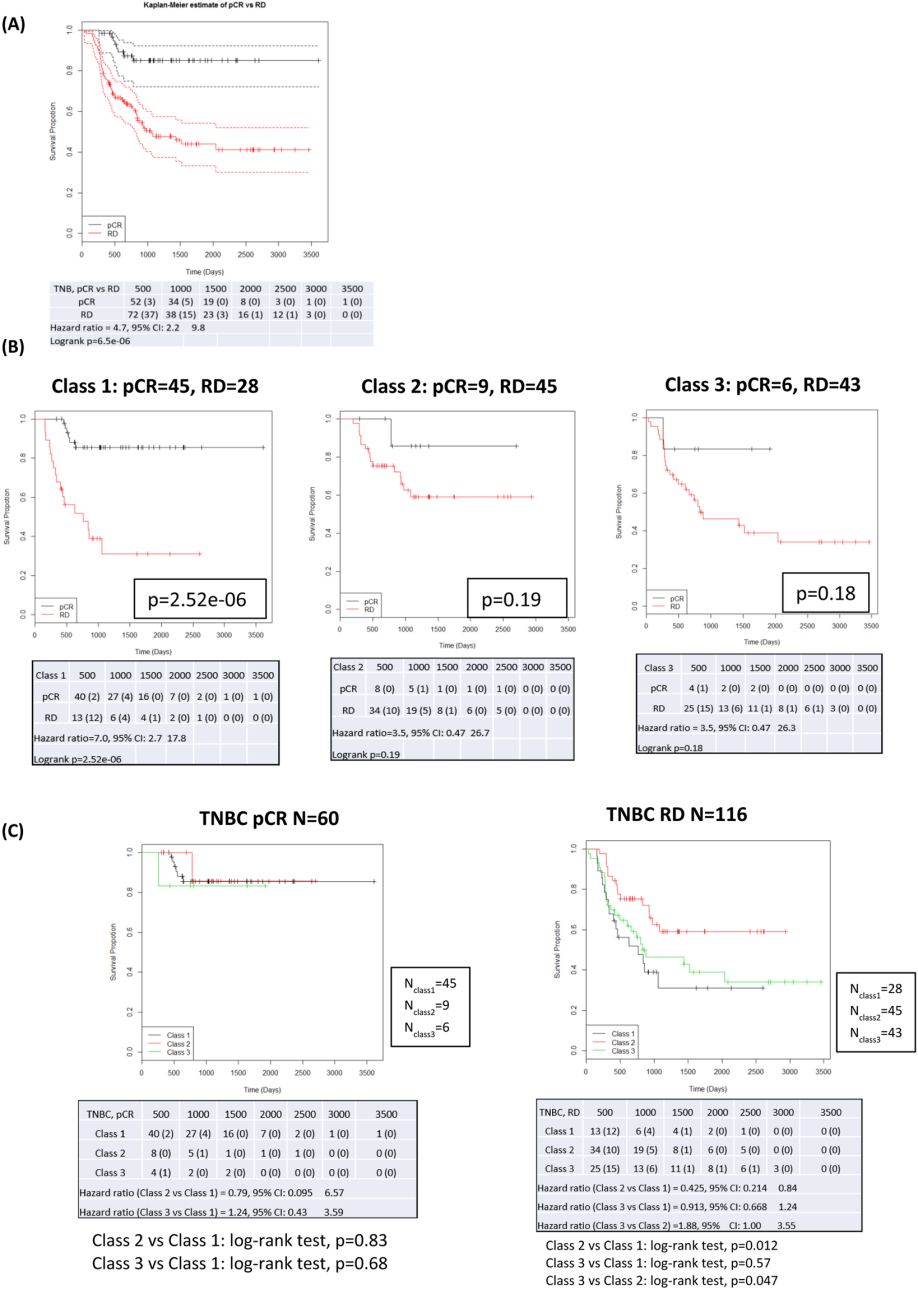
KM curves showing DRFS for TNBC patients over a maximum of 10 years of follow up. (**A**) The total population of TNBC patients prior to BA100 stratification is divided by actual pCR (black line) and RD (red line) (pCR = 60, RD = 116). The 95% confidence intervals are indicated by the dashed lines, and the hazard ratio, p-value, and numbers of patients and scored events at various days after treatment are shown below. (**B**) After BA100 stratification the DRFS KM curves for each Class of TNBC patients are displayed as above. (**C**) The left panel shows a comparison of DRFS for the pCR patients from all three classes. The black curve is class 1, the red curve is Class 2, and the green curve is Class 3. No significant differences are noted. The right panel shows a comparison of DRFS for the RD patients in all classes with the same curve colors as in the left panel. Here, there is a significant difference seen between Class 1 pCR and Class 2 RD patients (p = 0.012) and between Class 3 and Class 2 (p = 0.047).

Kaplan-Meier (KM) curves with up to 10 years DRFS are shown for TNBC patients achieving pCR or RD after NAC before BA100 stratification **(**Fig. 2A**)**, and with BA100’s Class 1, Class 2, and Class 3 stratification **(**Fig. 2B**)**. Achieving pCR was a surrogate marker for long term survival in all cases (hazard ratio = 4.7, p = 6.5e-06). Class 3 RD patients show a statistically significant worse prognosis in comparison with Class 2 RD patients (hazard ratio = 1.88, p = 0.047) (Fig. 2C right panel) showing that BA100 stratified RD patients into two groups with different risk profiles.

**Figure 3:**
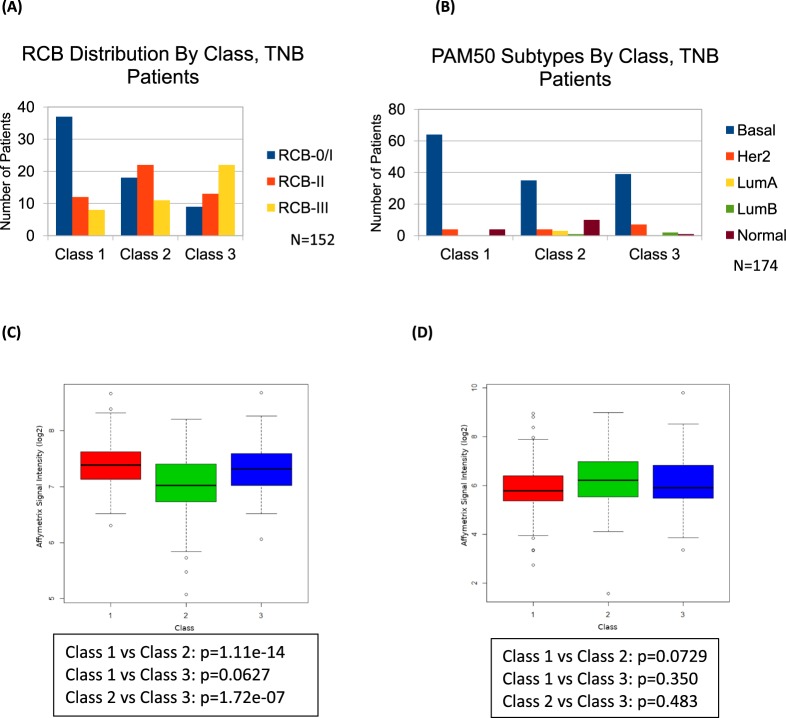
Clinical and gene expression comparisons with TNBC classes. (**A**) The distributions of RCB determined from the surgical specimen after NAC are shown for each BA100 class. Blue for RCB 0/I, orange for RCB-II, and gray for RCB-III. (**B**) The PAM50 classifications are shown for each BA100 class. (**C**) The expression levels of Ki-67 are shown as a box plot for Class 1 (red), Class 2 (green), and Class 3 (blue), with each box representing the interquartile range of gene expression, and a horizontal line inside showing the median expression. The p-values of pairwise t-tests are shown, indicating that Class 2 has significantly lower Ki-67 expression than the other two classes. (**D**) Expression of androgen receptor in TNBC tumors for each class is shown as in (**C)**.

To test the possibility that these classifications could also predict RCB^33^, a measure calculated after NAC and surgery, we determined the RCB distribution across the TNBC BA100 classification based on dataset annotations **(**Fig. 3A**)**. While RBC did not explain BA100 classification there were significant differences in RBC profile, with 65% of Class 1 comprised of RCB0/1, 35% and 43% of Class 2 being RCB 0/1 and RCB2 respectively, and 50% of Class 3 being RCB 3 (Pearson chi-squared p = 6.116e-06). Thus, BA100 can not only predict pCR or RD but can classify patients into classes presenting distinct RCB profiles from the initial patient biopsy, prior to any treatment.

We also tested if PAM50 molecular subtypes^34,35^ would show any association with the BA100 classes (Fig. 3B**)**. In TNBC, the expected large basal component is consistently dominant in all of BA100 classes ranging from 66% to 88% of the populations. BA100 Classes 2 and 3 show a discrete increase in numbers of normal and HER2 molecular subtypes respectively (Pearson chi-squared p = 0.002231).

Ki-67 expression showed highly significant differences between BA100 classes. In Fig. 3C, box plots show that while TNBC classes 1 and 3 have similar Ki-67 expression, each with median expression close to 7.4 and an interquartile range of about 7.0 to 7.6, class 2 shows significantly lower levels than the other classes (Class 2 vs Class 3 p = 1.72e-7), with a median expression of 7.0 and an interquartile range of 6.8 to 7.4. Androgen receptor expression was also assessed (Fig. 3D), and Class 2 had a higher median expression of AR, but it did not meet the p < 0.05 threshold of significance. The top portion of Supplemental Table 1 shows the distribution of various TNBC tumor parameters per class. There is no significant association between TNBC classes and histologic grade, clinical stage, T-Stage, or nodal status.

### Test of BA100 Against an External Validation Set

We analyzed an independent validation cohort of 304 patients treated with neoadjuvant T-FAC (demographics are shown in Supplemental Table 2) using the BA100 test. ER + /HER2- tumors comprised 44% of the cohort, TNBC tumors comprised 28%, and there was an overall pCR rate of 18.8% **(**Table 1 lower panel). Though the pCR rate is lower compared to the 519-member general population, we used the cohort to mimic real-life testing of sequential samples analyzed on a sample-by-sample basis without batch correction, as would be the case for use of BA100 in a clinical setting. BA100 correctly stratified 21.1% (12/57) of the pCR and 91.5% (226/247) of the RD patients in this cohort, for an overall accuracy of 78.3%. For the small TNBC subset, BA100 correctly stratified 30.0% (6/20) of the pCR and 86.2% (56/65) RD patients, for an overall accuracy of 72.9%. The prediction of RD patients in the validation cohorts had slightly better accuracy than that of the original cohort (91.5% vs 83.5%), but the accuracy of pCR predictions was much lower in the validation set (70.5% in training, 21.1% in validation). We think that the lack of batch correction in combining the three external validation data sets and the different composition of pCR vs RD, as well as Her2 patients may be contributors to the lower accuracy observed. Additional studies will be needed to further validate the BA100 test.

## Discussion

Achieving pCR is associated with significantly improved DRFS in TNBC patients, and previous work also supports the importance of achieving pCR for a variety of survival metrics, suggesting that pCR should be a major goal of NAC^10,33^. The benefit for TNBC patients is particularly strong as the risk functions modeled by the KM curves show that only 15% of TNBC patients with pCR are expected to suffer distant recurrences as compared to 60% of the RD patients over a 10-year span (Fig. 2A). There is also benefit in identifying those patients who will have RD with standard NAC, so the treatment can be altered to increase the chances of achieving pCR. These observations spurred us to develop a method to predict which patients are likely to experience pCR with the standard of care NAC, and alternatively which patients could be spared more aggressive chemotherapy regimens or be assigned to novel treatments in clinical trials (e.g. carboplatin, capecitabine)^36–38^.

We report here a gene profiling test using RNA classifiers on data from the initial biopsy to predict pCR or RD for breast cancer NAC. The BA100 model shows high negative predictive values for all subtypes of breast cancer. If BA100 was utilized to stratify patients prior to treatment, and only those predicted to have pCR were treated, pCR would then be 54% rather than 21.6% The ability to predict this response would be helpful in choosing standard options. Conversely, the ability to proactively identify a high percentage of TNBC RD patients with poor prognosis will allow physicians and patients to pursue more aggressive or targeted therapies at the beginning of treatment, rather than waiting for NAC treatment results.

Additionally, the further stratification of patients with residual disease into Class 2 and 3 with distinct biological features and significant differences in DRFS has not been detected in other recently proposed models of TNBC gene expression stratification^17,27^. The observation that Class 2 (lower risk RD) exhibits significantly lower expression of Ki-67 than the high-risk Class 3 or NAC-sensitive Class 1 suggests that while higher levels of proliferation may generate more patients with pCR after NAC, the tumors in Class 3 may be enriched for resistance and hence yield overall poorer outcomes. Though high levels of Ki-67 in unstratified patients were reported to respond better to NAC^39^, the BA100 classification system identifies high Ki-67 expressers which resist NAC and have worse outcomes than lower level Ki-67 expressers, demonstrating the value of stratifying patients. This study creates the rationale for further clinical validation and use of the test in research and development.

## Supplementary information

Supplementary information

## Data Availability

The datasets used and/or analyzed during the current study are available from the corresponding author on reasonable request.
