# Supplementary material for: A Predictor of Pathological Complete Response to Neoadjuvant Chemotherapy Stratifies Triple Negative Breast Cancer Patients with High Risk of Recurrence
Source: Sci Rep. 2019 Oct 16;9:14863. doi: 10.1038/s41598-019-51335-1 (PMC6795899; doi:10.1038/s41598-019-51335-1)
Supplement: Supplementary file 1 — Supplementary information [file 41598_2019_51335_MOESM1_ESM.pdf]

Title: A Predictor of Pathological Complete Response to Neoadjuvant Chemotherapy Stratifies Triple Negative Breast Cancer Patients with High Risk of Recurrence.

Authors:

Marcia V. Fournier Ph.D.[1*], Edward C. Goodwin Ph.D.[1], Joan Chen Ph.D.[2], John Obenauer Ph.D.[2], Susan H. Tannenbaum M.D.[3], Adam M. Brufsky M.D.[4]

1. Bioarray Genetics Inc. 400 Farmington Ave. Farmington CT 06032.
2. Rancho Biosciences. 16955 Via Del Campo #220, San Diego, CA 92127.
3. Division of Hematology/Oncology, UCONN Health Center. 263 Farmington Ave, Farmington, CT 06030.
4. Division of Hematology/Oncology, University of Pittsburg School of Medicine. 300 Halket St # 4628, Pittsburgh, PA 15213.

*Corresponding author

Corresponding email address: mvf@bioarray.us

Supplemental Table Legends:

Supplemental Table 1: The tumor characteristics are shown and segregated by BA100 class for the TNBC population (top) and for the four tumor subtypes in the total population (bottom). Some categories do not sum up to 177 data points as some patient data was incomplete. The tumor receptor subtypes in the entire population of 519 patients are shown at the bottom and separated by class.

Supplemental Table 2: The numbers of patients in different clinical categories is shown for the external validation data set.

Supplemental Table 3: The gene symbols, coefficients, intercepts, and threshold values are shown for Classifiers 1 and 2 in order of magnitude of the coefficients. Positive coefficients predict pCR and negative coefficients RD.

Supplemental Table 1: Tumor Characteristics by Class:

TNBC TUMORS:								
	Class I		Class II		Class III		Total	
**HISTOLOGIC GRADE**	N	%	N	%	N	%	N	%
1	0	0.0	1	1.9	0	0.0	1	0.6
2	9	12.9	5	9.6	9	18.8	23	13.5
3	60	85.7	46	88.5	39	81.3	145	85.3
4 (undetermined)	1	1.4	0	0.0	0	0.0	1	0.6
Total	70	100.0	52	100.0	48	100.0	170	100.0
**CLINICAL STAGE**	N	%	N	%	N	%	N	%
I	1	1.4	1	1.9	1	2.0	3	1.7
IIA	13	17.6	11	20.4	5	10.2	29	16.4
IIB	28	37.8	13	24.1	14	28.6	55	31.1
IIIA	17	23.0	14	25.9	13	26.5	44	24.9
IIIB	8	10.8	10	18.5	13	26.5	31	17.5
IIIC	7	9.5	5	9.3	0	0.0	12	6.8
Inflammatory	0	0.0	0	0.0	3	6.1	3	1.7
Total	74	100.0	54	100.0	49	100.0	177	100.0
**CLINICAL T STAGE**	N	%	N	%	N	%	N	%
T0	9	12.2	1	1.9	0	0.0	10	5.6
T1	6	8.1	6	11.1	7	14.3	19	10.7
T2	31	41.9	27	50.0	17	34.7	75	42.4
T3	20	27.0	11	20.4	13	26.5	44	24.9
T4	8	10.8	9	16.7	12	24.5	29	16.4
Total	74	100.0	54	100.0	49	100.0	177	100.0
**CLINICAL N STATUS**	N	%	N	%	N	%	N	%
N0	23	31.5	15	28.3	13	27.1	51	29.3
N1	30	41.1	23	43.4	12	25.0	65	37.4
N2	11	15.1	5	9.4	17	35.4	33	19.0
N3	9	12.3	10	18.9	6	12.5	25	14.4
Total	73	100.0	53	100.0	48	100.0	174	100.0
**RESPONSE TO NAC**	N	%	N	%	N	%	N	%
pCR	45	60.8	9	16.7	6	12.2	60	33.9
RD	29	39.2	45	83.3	43	87.8	117	66.1
Total	74	100.0	54	100.0	49	100.0	177	100.0
**PROSIGNA SUBTYPE**	N	%	N	%	N	%	N	%
Basal	65	89.0	35	66.0	39	79.6	139	79.4
Her2	4	5.5	4	7.5	7	14.3	15	8.6
LuminalA	0	0.0	3	5.7	0	0.0	3	1.7
LuminalB	0	0.0	1	1.9	2	4.1	3	1.7
Normal	4	5.5	10	18.9	1	2.0	15	8.6
Total	73	100.0	53	100.0	49	100.0	175	100.0
**TOTAL PATIENT POPULATION:**								
**TUMOR RECEPTOR SUBTYPE**	N	%	N	%	N	%	N	%
ER+/HER2-	51	34.9	191	72.1	49	45.4	291	56.1
TNBC	74	50.7	54	20.4	49	45.4	177	34.1
HER2+	13	8.9	13	4.9	7	6.5	33	6.4
ER-/HER2-/PR+	8	5.5	7	2.6	3	2.8	18	3.5
Total	146	100.0	265	100.0	108	100.0	519	100.0

Suppl. Table 2: Demographics of External Validation Set

	Patients	Age		Clinical T Stage					Clinical Nodal Status				Clinical Stage						Histological Grade		
		Median	Range	T0	T1	T2	T3	T4	N0	N1	N2	N3	IA	IIA	IIB	IIIA	IIIB	IIIC	1	2	3
**Total Population:**																					
pCR	57	53	28-71	1	7	33	6	10	11	18	7	6	0	2	13	2	2	0	2	7	6
RD	247	50	26-79	2	14	126	54	48	62	63	30	24	0	11	15	14	15	4	11	47	8
**TNBC:**																					
pCR	19	56	28-70	0	1	14	2	2	2	5	2	0	0	2	13	2	2	0	0	4	6
RD	62	52	29-74	0	2	25	17	17	12	19	10	4	0	11	15	14	15	4	1	10	5
**ER+/HER2-:**																					
pCR	9	46	36-61	0	1	5	0	3	1	1	2	0	0	3	3	0	3	0	2	3	0
RD	122	49	26-79	2	9	79	18	14	27	24	9	11	1	32	45	14	13	11	10	37	3

Suppl. Table 3: Genes and Formula comprising BA100

Classifier 1

coefficient	Gene Symbol
0.1360	DEPDC1
0.1043	HAT1
0.0821	H2AFZ
0.0685	ODC1
0.0386	RFWD3
0.0351	DROSHA
0.0245	EHD1
0.0180	CENPX
0.0123	VRK1
-0.0167	LAMC2
-0.0185	PLEK2
-0.0198	WHSC1
-0.0260	RRM2
-0.0261	SRCAP
-0.0519	PSMB2
-0.0578	NOP10
-2.065	Intercept
0.207	Threshold

Classifier 2

coefficients	Gene Symbol
0.1882	ACTB
0.1514	H2AFV
0.1058	CENPX1
0.0773	EHF
0.0474	ELN
0.0363	CSN3
0.0302	HLA-DPA1
0.0292	IGF2BP2
0.0128	NPY1R
-0.0040	MSLN
-0.0194	BCAR3
-0.0210	PTPN20B
-0.0218	TDRD12
-0.0282	S100P
-0.0378	PTH2R
-0.0414	LYPLA1
-5.00	Intercept
0.2810	Threshold
